# Integrality of the therapeutic and pharmaceutical care: a necessary debate

**DOI:** 10.11606/S1518-8787.2017051000185

**Published:** 2017-12-04

**Authors:** Fabiola Sulpino Vieira

**Affiliations:** I Instituto de Pesquisa Econômica Aplicada. Diretoria de Estudos e Políticas Sociais. Brasília, DF, Brasil

**Keywords:** Pharmaceutical Services, Legislation & Jurisprudence, Integrality in Health, Unified Health System, Right to Health, Assistência Farmacêutica, Legislação & Jurisprudência, Integralidade em Saúde, Sistema Único de Saúde, Direito à Saúde

## Abstract

The controversy surrounding the different interpretations on the integrality of therapeutic and pharmaceutical care has led to the delimitation of its scope by a law, but the issue has not been completely pacified. As a contribution to this debate, we aim to discuss the challenges to ensure the integrality of the therapeutic and pharmaceutical care, based on a conceptual approach on the meanings of integrality in the Brazilian Unified Health System (SUS). We identified important challenges to ensure the integrality of the therapeutic and pharmaceutical care in the SUS. These challenges are related to professional practices, the organization of actions and services, and the governmental response to health problems or to the treatment of specific population groups. For this end, governments need to carry out structuring actions and be efficient in using available resources so that existing problems can be overcome.

## RESUMO

A polêmica em torno de diferentes interpretações sobre a integralidade da assistência terapêutica e farmacêutica levou à delimitação de sua abrangência por meio de uma lei, mas sem que a questão tenha sido completamente pacificada. Como contribuição a esse debate, o objetivo deste texto é o de discutir os desafios à garantia da integralidade da assistência terapêutica e farmacêutica, a partir de uma abordagem conceitual sobre os sentidos da integralidade no Sistema Único de Saúde (SUS). Identificam-se desafios importantes para a garantia da integralidade da assistência terapêutica e farmacêutica no SUS. Esses desafios estão relacionados às práticas profissionais, à organização das ações e serviços e à resposta governamental para problemas de saúde ou para tratamento de grupos populacionais específicos. Isso exige dos governos ações estruturantes e eficiência no uso dos recursos disponíveis, a fim de que os problemas existentes sejam superados.

## INTRODUCTION

Access to essential drugs is considered an important public policy tool to improve the quality of life of populations, and the appropriate use of these drugs is one of the most cost-effective components of health care^a^. The access to them demands the articulation of a set of actions and services within the health system, as well as broader governmental actions, such as the promotion of research, development of drugs, and the sanitary and economic regulation of the market.

In Brazil, this set of actions and services was called pharmaceutical care^b^ and gained prominence in the national public debate since the mid-2000s, when extended judicial decisions determined the supply of medicines to citizens by the managers of the Brazilian Unified Health System (SUS). The issue of the integrality of therapeutic care (ITC) is at the heart of this debate, with different interpretations on its impacts, leading to the delimitation of its scope, without solving the issue^c^.

Less discussed is the integrality of the pharmaceutical care (IPC). What it means, what is its scope, and how to ensure them are issues that need to be analyzed and this seems to be an opportune moment, since ideas are presented to replace the dispensation of drugs in the primary health care in the SUS by private pharmaceutical establishments, in a model similar to that of the Popular Pharmacy Program.

As a contribution to this debate, we aim to discuss the challenges to ensure the integrality of the therapeutic and pharmaceutical care (ITPC), based on a conceptual approach on the meanings of integrality of the health care in the SUS.

### Universality and Integrality of the Access to Health Actions and Services

The Brazilian Federal Constitution establishes the integrality of care in the SUS as one of the guidelines of the system, focusing on preventive activities and without impairment of the care services^d^. Subsequently, it was defined that comprehensive therapeutic care, including pharmaceutical care, is included in the field of activity of the SUS^e^.

Mattos^1^ ^,^ ^2^ discusses three sets of meanings related to integrality, regarding: (i) the practices of health professionals, in which there is a concern to discern the needs of users, (ii) the organization of health services and practices, because they must be organized to achieve a broader understanding of the needs of the population, the link between prevention and care, and the continuous organization of work processes, and (iii) government responses to certain health problems or the needs of specific groups.

Under the SUS, this broader understanding of integrality appears to be a reality. However, the phenomenon of health judicialization has shown that, in a more general social context, this is not a hegemonic interpretation^3^, something that has worried health managers by the expressive number of lawsuits^f^.

It is clear that the increase in the legal demands for drugs shows greater access of the population to the Judicial Branch and greater awareness about the right to health^4^. It also indicates that a significant part of the requests refers to pharmaceutical products that are present in public policies, but to which persons have difficulty accessing for different reasons^5^. However, we can also observe that if judicial decisions do not consider public policies and principles of the SUS, they have great potential to disorganize it^6^ and increase inequity in access to health services and actions^7^.

The ITC cannot be interpreted as everything for everyone, as an indiscriminate right to any technology. In the national or international pharmaceutical market, many drugs overlap, have less therapeutic potential, or present greater risks compared to others already available in the SUS, and they can be much more expensive.

The controversy surrounding the understanding on the ITC and the judicialization of the health sector motivated the approval of Law 12,401, defining integrality as the ensuring of the access to drugs present in clinical protocols and in drug relations elaborated by the managers of the SUS^c^. However the passage of this law does not solve the issue. First, because it stresses the principle of universality, as in the case of drugs not incorporated into the SUS for the treatment of rare diseases^8^. All citizens have the right to universal and equal access to policies for the promotion, protection, and recovery of their health and this principle is understood as that which is common to all^9^. Second, because the Federal Supreme Court has already established jurisprudence on the subject, and the understanding is that: (i) the supply of drugs without registration in the National Health Agency (ANVISA) is only admitted under exceptional conditions, (ii) the separation of the Powers prohibits the possibility of a law replacing the registration of the drug, which is typical of the Executive Branch, (iii) it is necessary to observe, in principle, the protocols, (iv) however, universality must be ensured, and the Judicial Branch may intervene in cases of omission of the Executive Branch, and (v) the subjective public right to health represents an indissociable prerogative of the right to life^10^. As we can see, the ITPC involves a set of complex challenges for the Government.

### Challenges to Ensure the ITPC in the SUS

The SUS also faces important problems to ensure the access and rational use of drugs to the population. Some evaluations of the pharmaceutical care that also bring the discussion of integrality show that many problems still need to be addressed^11^ ^-^ ^15^.

As for professional practices, the challenges of the ITPC are the training and qualification of pharmacists for the practice of clinical pharmacy, the recognition of their role in health teams, and the integrated action of other health professionals. Some initiatives have been successful in this sense^16^ ^,^ ^g^, but the dimensions of the SUS impose the need to implement more structuring measures in this regard, with a focus on promoting the rational use of drugs.

From the point of view of the organization of services, many municipalities also need to ensure: a) the timing of the procurement and distribution of drugs, b) the integration and communication between pharmacies, c) the availability of pharmaceutical products, d) the information to patients about the organization of services, e) the practice of the clinical pharmacy, f) the adequate infrastructure, including computerization of pharmacies and supply centers, in addition to their connection with other health services, and g) the economicity, both in relation to drugs and the model of management of pharmaceutical care.

Regarding this last aspect, we have observed new organizational arrangements that bring advantages and disadvantages to the access to drugs, their rational use, and the economics in the SUS (Box).

**Box:** Models of management of pharmaceutical care and possible consequences for the access to drugs and public administration.

Models of management of pharmaceutical care	Example	Access to drugs	Some possible difficulties from the perspective of the public administration
State public management	State public pharmaceutical care (most of the Brazilian municipalities)	It depends on the performance of the Health Department team in the management of the logistics component and the regular financing of the procurements	Management of procurement processes Difficulties in complying with distribution schedules Lack of drugs in health units
Integrated public and private mix	Management of health units, including pharmacies, by social organizations (OSS), but the global management of pharmaceutical care is under the responsibility of the public administration	It depends on the performance of the Health Department team in the management of the logistics component of pharmaceutical care and the routine financing of the procurements	The problems mentioned above may arise because the logistical component of pharmaceutical care falls under the responsibility of the public administration Difficulties in coordinating the actions of the technical component (the OSS is responsible for the management of the health unit and the pharmacists are hired by it)
Segmented public and private mix	Public or state public pharmaceutical care from the OSS coexisting with the Popular Pharmacy Program	It is facilitated by the supply of drugs by the private network of pharmaceutical establishments (drugstores)	Overlapping programs with different costs for the same paying source and induction of the market to ensure the offering only by the program that is more profitable Costs may be higher than in other models Difficulties in the regulation and control of expenses Disarticulation between the technical and logistic components of pharmaceutical care Pharmacotherapeutic monitoring by the public administration is impaired, unless mechanisms are created to link the health services of the SUS to the private network Fragmentation of the care may be greater

Obviously, the issues surrounding models and their results are not exhausted by the few variables presented in the Box, which illustrates the potential fragmentation of services and care. It is worth noting that even if a particular health department decides to adopt a privatized model of dispensing drugs, it would still have to maintain services under its management since several drugs of the National Essential Medicine List (RENAME) are not marketed by drugstores, and also that the fragmentation of the care occurs in the SUS, but it can be aggravated in mixed models.

In addition, we highlight that the costs may be higher for the Government in the segmented model, in which drugstores act in the dispensing of drugs and are reimbursed by the Government for both the product and the service. Studies comparing the costs of offering drugs with the public pharmaceutical care to those of the Popular Pharmacy Program concluded that the costs are lower in the SUS^17^ ^,^ ^18^.

In the federal government, great concern about the growth of spending with the Popular Pharmacy Program has motivated the adoption of measures to regulate access to drugs. In real terms, expenditures rose 667% between 2010 and 2015, with a decrease between 2015 and 2016, when they went from R$3 billion to R$2.7 billion in 2016, from the negotiation of prices made by the Ministry of Health (Figure).

**Figure:**
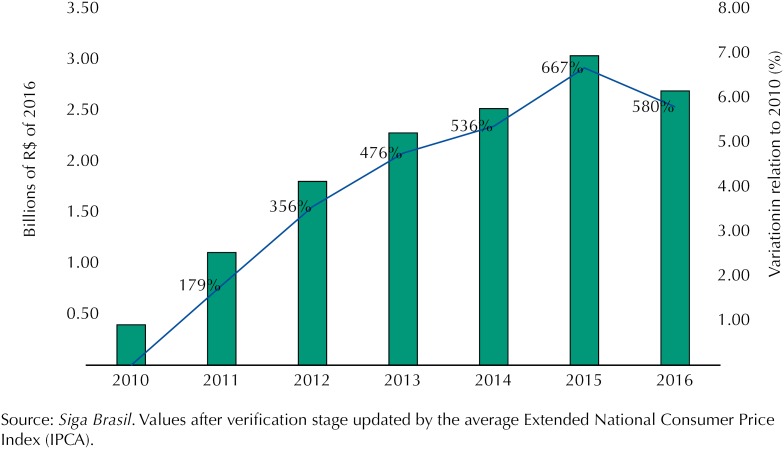
Spending of the Ministry of Health with drugs under the Popular Pharmacy Program, 2010-2016.

Source: *Siga Brasil*. Values after verification stage updated by the average Extended National Consumer Price Index (IPCA).

In the same period, the Components of the Pharmaceutical Care Financing Block had a much more modest evolution or even a decrease. In 2016 values, the Basic Component went from R$1.6 billion to R$1.2 billion (decrease of 25%), the Strategic Component went from R$3.2 billion to approximately R$5 billion (increase of 53%), and the Specialized Component went from R$4.9 billion to R$6.7 billion (increase of 36%).

The availability of financial resources to ensure access to drugs matters a lot, as does their use. For this reason, since the 1970s, the World Health Organization has included in the concept of essential drugs and the rational use of drugs the relevance of comparing the effectiveness of products to the costs and ensuring the lowest cost for patients and their communities^h^ ^,^ ^i^. That is why we use economic evaluations, which compare the costs and results of each policy or technology option before its implementation or incorporation.

Resources are in fact scarce to meet the many needs of the population and therefore need to be used rationally, which again refers to the discussion about integrality in the SUS. There seems to be a consensus among Brazilian bioethicists that the universal right to health is a fundamental principle but that integrality must be regulated^19^. The allocation of scarce resources should be done with popular participation, considering the facts, principles, values, emotions, ideas, and beliefs of society^20^.

Regarding government responses to health problems and the needs of specific groups, we also identified important gaps, especially regarding the treatment of some rare diseases and alternatives for patients who cannot use the drugs listed in RENAME. In addition, insufficient financing of the SUS, sanitary and economic regulation, aging of the population, release of new drugs, management of health technologies, and financial sustainability of the system are important issues for public managers.

Thus, there are still major challenges to ensure the ITPC in the SUS, either in the sense of professional practices, the organization of actions and services, or in the governmental response to health problems or specific groups. This requires structuring actions and efficient use of available resources by Governments.

Regarding the delimitation of the access to drugs, we should consider the establishment of criteria for the regulation of the access of patients in exceptional situations that are not in the lists of the SUS. However, before that, some consensus on this subject should be established. It is unreasonable for the SUS to offer experimental drugs that are not proven to be effective and safe or that can be replaced by other drugs already available in the lists. In addition, the cost of treatment also needs to be considered. If the issues involving the allocation of scarce resources from society ethically require popular participation, would it not be the time to hold a national pharmaceutical care conference to discuss these issues?

## Footnotes

a Organización Mundial de la Salud. Selección de medicamentos esenciales. Ginebra: OMS; 2002 [cited 2017 Sep 30]. (Perspectivas Políticas de Medicamentos de la OMS, 4). Available from: http://apps.who.¡nt/med¡c¡nedocs/es/p/printable.html

b Ministerio da Saúde (BR), Secretaria de Políticas de Saúde, Departamento de Formulação de Políticas de Saúde. Política Nacional de Medicamentos. Brasília (DF); 2001 [cited 2017 Sep 30]. (Série C. Projetos, Programas e Relatórios, 25). Available from: http://bvsms.saude.gov.br/bvs/publicacoes/politica_medicamentos.pdf

c Brasil. Lei n° 12.401, de 28 de abril de 2011. Altera a Lei n° 8.080, de 19 de setembro de 1990, para dispor sobre a assistência terapêutica e a incorporação de tecnologia em saúde no âmbito do Sistema Único de Saúde - SUS. *Diário Oficial União.* 29 abr 2011; Seção 1:1-2.

d Brasil. Constituição (1988). Constituição da República Federativa do Brasil. Brasília (DF); 1988 [cited 2017 Oct 19]. Available from: http://www.planalto.gov.br/ccivil_03/constituicao/constituicao.htm

e Brasil. Lei n° 8.080, de 19 de setembro de 1990. Dispõe sobre as condições para a promoção, proteção e recuperação da saúde, a organização e o funcionamento dos serviços correspondentes e dá outras providências. *Diário Oficial União.* 20 set 1990; Seção 1:1-5.

f Asensi FD, Pinheiro R. Judicialização da saúde no Brasil: dados e experiências. Brasília (DF): Conselho Nacional de Justiça; 2015 [cited 2017 Sep 30]. Available from: http://www.cnj.jus.br/files/conteudo/destaques//arquivo/2015/06/6781486daef02bc6ec8c1e491a565006.pdf

g Ministério da Saúde (BR), Secretaria de Ciência, Tecnologia e Insumos Estratégicos, Departamento de Assistência Farmacêutica e Insumos Estratégicos, Coordenação-Geral de Assistência Farmacêutica Básica. Resultados do projeto de implantação do cuidado farmacêutico no Município de Curitiba. Brasília (DF); 2015 [cited 2017 Sep 30]. (Cuidado Farmacêutico na Atenção Básica, caderno 4). Available from: http://bvsms.saude.gov.br/bvs/publicacoes/resultado_projeto_implantacao_cuidados_farmaceuticos.pdf

h Organización Mundial de la Salud. Selección de medicamentos esenciales. Ginebra: OMS; 2002. (Perspectivas políticas de medicamentos de la OMS, 4.)

i World Health Organization. Promoting rational use of medicines: core components. Geneva: WHO; 2002. (WHO Policy Perspectives on Medicines, 5).
